# Calls to Poison Centers for Exposures to Electronic Cigarettes — United States, September 2010–February 2014

**Published:** 2014-04-04

**Authors:** Kevin Chatham-Stephens, Royal Law, Ethel Taylor, Paul Melstrom, Rebecca Bunnell, Baoguang Wang, Benjamin Apelberg, Joshua G. Schier

**Affiliations:** 1 EIS officer, CDC; 2 Division of Environmental Hazards and Health Effects, National Center for Environmental Health, CDC; 3 Office on Smoking and Health, National Center for Chronic Disease Prevention and Health Promotion, CDC; 4 Center for Tobacco Products, Food and Drug Administration

Electronic nicotine delivery devices such as electronic cigarettes (e-cigarettes) are battery-powered devices that deliver nicotine, flavorings (e.g., fruit, mint, and chocolate), and other chemicals via an inhaled aerosol. E-cigarettes that are marketed without a therapeutic claim by the product manufacturer are currently not regulated by the Food and Drug Administration (FDA) (1).* In many states, there are no restrictions on the sale of e-cigarettes to minors. Although e-cigarette use is increasing among U.S. adolescents and adults (2,3), its overall impact on public health remains unclear. One area of concern is the potential of e-cigarettes to cause acute nicotine toxicity (4). To assess the frequency of exposures to e-cigarettes and characterize the reported adverse health effects associated with e-cigarettes, CDC analyzed data on calls to U.S. poison centers (PCs) about human exposures to e-cigarettes (exposure calls) for the period September 2010 (when new, unique codes were added specifically for capturing e-cigarette calls) through February 2014. To provide a comparison to a conventional product with known toxicity, the number and characteristics of e-cigarette exposure calls were compared with those of conventional tobacco cigarette exposure calls.

An e-cigarette exposure call was defined as a call regarding an exposure to the e-cigarette device itself or to the nicotine liquid, which typically is contained in a cartridge that the user inserts into the e-cigarette. A cigarette exposure call was defined as a call regarding an exposure to tobacco cigarettes, but not cigarette butts. Calls involving multiple substance exposures (e.g., cigarettes and ethanol) were excluded. E-cigarette exposure calls were compared with cigarette exposure calls by proportion of calls from health-care facilities (versus residential and other non–health-care facilities), demographic characteristics, exposure routes, and report of adverse health effect. Statistical significance of differences (p<0.05) was assessed using chi-square tests.

During the study period, PCs reported 2,405 e-cigarette and 16,248 cigarette exposure calls from across the United States, the District of Columbia, and U.S. territories. E-cigarette exposure calls per month increased from one in September 2010 to 215 in February 2014 (Figure). Cigarette exposure calls ranged from 301 to 512 calls per month and were more frequent in summer months, a pattern also observed with total call volume to PCs involving all exposures (5).

E-cigarettes accounted for an increasing proportion of combined monthly e-cigarette and cigarette exposure calls, increasing from 0.3% in September 2010 to 41.7% in February 2014. A greater proportion of e-cigarette exposure calls came from health-care facilities than cigarette exposure calls (12.8% versus 5.9%) (p<0.001). Cigarette exposures were primarily among persons aged 0–5 years (94.9%), whereas e-cigarette exposures were mostly among persons aged 0–5 years (51.1%) and >20 years (42.0%). E-cigarette exposures were more likely to be reported as inhalations (16.8% versus 2.0%), eye exposures (8.5% versus 0.1%), and skin exposures (5.9% versus 0.1%), and less likely to be reported as ingestions (68.9% versus 97.8%) compared with cigarette exposures (p<0.001).

Among the 9,839 exposure calls with information about the severity of adverse health effects, e-cigarette exposure calls were more likely to report an adverse health effect after exposure than cigarette exposure calls (57.8% versus 36.0%) (p<0.001). The most common adverse health effects in e-cigarette exposure calls were vomiting, nausea, and eye irritation. One suicide death from intravenous injection of nicotine liquid was reported to PCs.

Calls about exposures to e-cigarettes, which were first marketed in the United States in 2007, now account for 41.7% of combined monthly e-cigarette and cigarette exposure calls to PCs. The proportion of calls from health-care facilities, age distribution, exposure routes, and report of adverse health effects differed significantly between the two types of cigarette.

This analysis might have underestimated the total number of e-cigarette and cigarette exposures for several reasons. Calls involving e-cigarettes or cigarettes and another exposure were excluded, and the code indicating a case of e-cigarette exposure might have been underused initially. In addition, health-care providers, including emergency department providers, and the public might not have reported all e-cigarette or cigarette exposures to PCs. Given the rapid increase in e-cigarette-related exposures, of which 51.1% were among young children, developing strategies to monitor and prevent future poisonings is critical. Health-care providers; the public health community; e-cigarette manufacturers, distributors, sellers, and marketers; and the public should be aware that e-cigarettes have the potential to cause acute adverse health effects and represent an emerging public health concern.

## Figures and Tables

**FIGURE:**
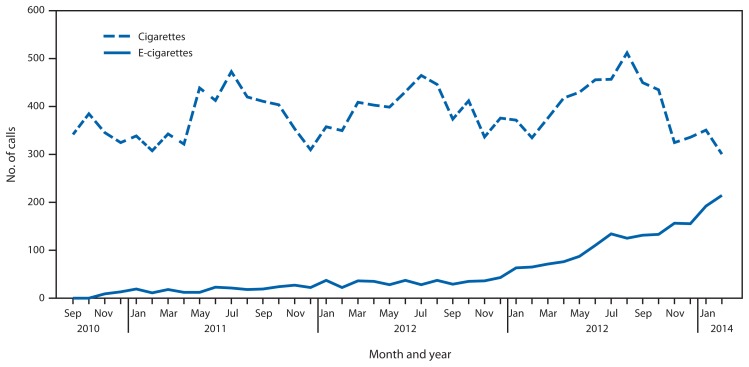
Number of calls to poison centers for cigarette or e-cigarette exposures, by month — United States, September 2010–February 2014

## Footnotes

* Currently, e-cigarettes and their components, such as the nicotine they contain, that are intended for therapeutic purposes (e.g., for smoking cessation) are drug/device combination products. When they are marketed for therapeutic purposes they are regulated by the FDA’s Center for Drug Evaluation and Research. FDA’s Center for Tobacco Products currently regulates cigarettes, cigarette tobacco, roll-your-own tobacco, and smokeless tobacco. FDA has stated its intention to issue a proposed rule extending FDA’s tobacco product authorities beyond these products to include other products like e-cigarettes not intended for therapeutic purposes.
